# Genome-Wide Identification and Characterization of C3H-ZFP Genes and Their Expression Under Salt and Cadmium Stress Conditions in Soybean

**DOI:** 10.3390/cimb48030287

**Published:** 2026-03-08

**Authors:** Intikhab Alam, Khadija Batool, Hui-Cong Wang, Fang Qiao

**Affiliations:** 1 School of Food and Drugs, Shenzhen Polytechnic University, Shenzhen 518055, China; 2 Key Laboratory of Biology and Genetic Improvement of Horticultural Crops-South China College of Horticulture, South China Agricultural University, Guangzhou 510642, China; 3 Key Laboratory of Ministry of Education for Genetics, Breeding and Multiple Utilization of Crops, College of Agriculture, Fujian Agriculture and Forestry University, Fuzhou 350002, China

**Keywords:** soybean, C3H-ZFP genes, phylogenetic classification, functional divergence, gene expressions, salt stress, cadmium stress

## Abstract

Zinc finger proteins (ZFPs) are a diverse group of plant transcription factors essential for regulating development, signaling, and stress responses. In this study, we performed a genome-wide identification and integrative analysis of 140 C3H-type zinc finger transcription factor genes in the soybean genome, exhibiting an uneven distribution across all 20 chromosomes. These C3H-ZFPs contained one (37), two (58), three (19), four (7), five (17), or six (2) C3H domains and were classified into 14 subsets based on their domain architecture. All C3H genes encoding proteins harbored the conserved C3H-ZFP domain and displayed various physicochemical characteristics. Phylogenetic analysis grouped them into 10 clades, closely related to other species like Arabidopsis, rice and alfalfa. Promoter analysis revealed cis-elements associated with stress response (~39.1%), light response (~37.3%), phytohormones (~18.5%), and development (~4.97%). Duplication analysis revealed 78 pairs of segmental and eight tandem duplication events, with purifying selection indicated by Ka/Ks (nonsynonymous/synonymous) ratios, indicating that these C3H-ZFP duplicates were largely maintained under purifying selection. A total of 388 miRNAs from 196 gene families were predicted to target 140 C3H-ZFP genes, with most enriched miRNAs targeting C3H-ZFP genes, including the miR156, miR395, and miR396 families. Transcription factor binding sites for MYB, AP2, MIKC_MADS, BBR-BPC, ERF, C2H2, and Dof were found upstream of most C3H-ZFP genes. RNA-Seq and qRT-PCR analyses showed tissue-specific expression and stress-responsive expression patterns, with several C3H-ZFP genes, especially *GmC3H1*, *GmC3H63*, *GmC3H124*, and *GmC3H127*, being significantly upregulated under abiotic stress conditions. Together, these results provide a comprehensive overview of soybean C3H-ZFP genes and identify promising candidates for future functional studies on development and abiotic stress adaptation.

## 1. Introduction

Zinc finger proteins (ZFPs) are one of the largest and most functionally diverse families of transcription factors in plants. These proteins are classified into 10 main types based on the number and organization of cysteine (Cys) and histidine (His) residues as well as the spacing of amino acid residues between them. The major classes include C2C2, C2HC, C2HC5, C2H2, C3H, C3HC4, C4, C4HC3, C6, and C8 [1]. Among these, zinc finger proteins (C3H-ZFPs) form a distinct subgroup defined by zinc finger motifs containing three cysteine residues and one histidine residue, which coordinate a zinc ion. This structural motifi is evolutionarily conserved across all eukaryotic organisms and exhibits a characteristic consensus sequence initially described as C-X_6–14_-C-X_4–5_-C-X_3_-H, where “X” denotes any amino acid [2,3]. However, subsequent refinements have expanded the definition to C-X_4–17_-C-X_4–6_-C-X_3_-H, reflecting broader variability in the inter-residue spacings [4].

C3H-ZFPs typically function through interactions with nucleic acids, including both RNA and DNA, and participate in diverse physiological processes. In plants, these proteins have been implicated in transcriptional regulation, RNA metabolism, hormone signaling, immune responses, and adaptation to environmental cues [5,6]. Functional studies have established the involvement of C3H-type proteins in photoperiod sensitivity, hormone-mediated signaling pathways, and resistance to abiotic stressors, including drought, salinity and pathogenic infection [7]. The advent of high-throughput genomics and transcriptomics has enhanced the finding of novel C3H gene family members, contributing to a deeper understanding of their regulatory roles in plant growth and adaptation mechanisms [8].

Extensive characterization of C3H genes has been reported in several model and crop plants, including *Arabidopsis thaliana* [9], rice (*Oryza sativa*) [9], maize (*Zea mays*) [10], alfalfa (*Medicago sativa*) [11], citrus (*Citrus sinensis*) [12], and poplar (*Populus trichocarpa*) [13]. Cross-species studies have validated the conserved functions of C3H genes. In *A. thaliana*, overexpression of *AtC3H17*, *AtC3H29*, and *AtC3H47* has been associated with enhanced salt stress tolerance while *AtC3H49* contributes to improved resistance to both salt and drought stress [14,15]. Similarly, expression of *GhZFP1* in transgenic tobacco has been shown to strengthen ionic balance under salt stress by regulating sodium and potassium ion transport [16]. In *Brassica oleracea*, *BoC3H* overexpression led to increased salinity tolerance [17], while *PeC3H74*-overexpressing Arabidopsis plants exhibited increased drought tolerance compared to wild-type controls [18]. In addition to abiotic stress responses, C3H-ZFPs are involved in biotic stress defense and developmental regulation. In cotton (*Gossypium hirsutum*), GhZFP1 interacts with GhZIRD21A and GhZIPR5, enhancing resistance against fungal pathogens [16]. In *A. thaliana*, *C3H15* negatively regulates cell elongation by interfering with brassinosteroid signaling [19], while functioning in secondary cell wall formation [20]. In rice, *OsLIC* and *OsDOS* modulate brassinosteroid responses and contribute to architectural development and the delay of leaf senescence, respectively [9,21]. Additionally, *ZmC3H9* in maize is involved in the biosynthesis of phenolic compounds [22], and *GhTZF1* regulates leaf senescence in cotton [23]. During embryogenesis in *A. thaliana*, the embryo-specific gene PEI1 (Plant Embryo Induction 1) is expressed predominantly in the apical region of the developing embryo and is essential for normal development [24]. Despite these advances, recent studies have demonstrated that C3H-type zinc finger proteins play crucial roles in regulating plant growth and development, as well as in mediating responses to various environmental stresses. Although the functions of the C3H gene family have been extensively investigated in several model and crop species, their systematic identification, structural characterization, and functional analysis in soybean (*Glycine max*) remain limited. Given the agronomic importance of soybean as a major global source of plant protein and oil, a comprehensive investigation of its C3H gene family is both timely and necessary.

To address this gap, the present study conducted a systematic identification and analysis of C3H genes in soybean to better understand their genomic organization, evolutionary relationships, and potential involvement in stress responses. We analyzed the genomic distribution, phylogenetic relationships, conserved motifs, and evolutionary features of soybean C3H genes and compared them with their counterparts in Arabidopsis. Our results revealed soybean-specific C3H-ZFP subfamilies as well as conserved orthologous relationships between C3H genes in soybean and Arabidopsis, providing insights into the evolutionary diversification of this gene family. Expression analysis identified several GmC3H genes with tissue-specific and stress-responsive expression patterns, suggesting potential roles in soybean growth and stress adaptation. These findings offer valuable information on the evolutionary trajectory of the C3H gene family in soybean and establish a foundation for future functional studies aimed at improving stress tolerance.

## 2. Materials and Methods

### 2.1. Plant Materials and qRT-PCR Analysis

Seeds of the soybean (*G. max* [L.] Merr.) cultivar Williams 82, obtained from Guangdong National Center for Soybean Improvement, South China Agricultural University, Guangzhou, China, were used in this study. Seeds were surface-sterilized through immersion in a 1% sodium hypochlorite (NaClO) for five minutes, followed by gentle shaking and extensive rinsing with deionized water (ddH_2_O) to remove any residual disinfectant. Next, the seeds were sown in containers filled with a 1:1 mixture of soil and sand, providing a suitable growth medium. Plants were grown in containers placed in a controlled growth chamber under 16/8 h light/dark conditions, with a temperature of 22 °C and relative humidity set between 65 and 70%.

To investigate the potential roles of GmC3H-ZFP genes in mediating plant responses to cadmium (Cd) and salt (NaCl) stress, 7-day-old soybean seedlings were exposed to high concentrations of Cd: 0.05 mM Cd (CdCl2) and 250 mM NaCl [25,26,27,28,29]. The samples were collected at 0 (untreated control), 1, 3, and 6 h after treatment to assess temporal changes in gene expression under stress conditions. These time points were selected to evaluate early and progressive transcriptional responses. For RNA extraction, root tissue (~0.3 cm in length) from each soybean seedling was immediately frozen in liquid nitrogen.

Total RNA was isolated using an RNA isolation kit (OMEGA Bio-Tek, Guangzhou, China). Each treatment condition included three biological replicates to ensure data reliability. RNA integrity was assessed through agarose gel electrophoresis, whereas its concentration and purity were quantified using a NanoDrop 2000 spectrophotometer (Thermo Fisher Scientific, Waltham, MA, USA). First-strand cDNA synthesis was performed using 1 µg of total RNA from all samples and the PrimeScript™ RT kit (Takara, Dalian, China). The cDNA products were diluted at a 1:20 ratio and stored at −20 °C for following analysis. Primers specific to the GmC3H-ZFP genes were designed using the Primer3Plus online software (https://www.primer3plus.com accessed on 1 April 2025). The actin gene was employed as internal reference for normalization of gene expression levels (Table S9). qRT-PCR was employed to assess relative gene expression using the CFX-Bio-Rad RT System (Bio-Rad, Hercules, CA, USA). Data were analyzed using the 2^−ΔΔCt^ method, with normalization to the housekeeping gene actin. Expression levels of GmC3H-ZFP genes were compared across different treatment durations and stress exposure conditions.

### 2.2. Identification and Characterization of Soybean C3H-ZFPs

To identify C3H-ZFPs homologous in soybean, we initially retrieved the complete set of annotated C3H-ZFP sequences from Arabidopsis via the TAIR database (https://www.arabidopsis.org accessed on 1 April 2025). These sequences served as reference queries for BLASTp (v13) searches conducted against the *G. max* genome using the Phytozome v13 database (https://phytozome-next.jgi.doe.gov/ accessed on 1 April 2025), employing an E-value threshold of <1 × 10^−5^ to identify putative homologous sequences [30]. All candidate soybean C3H-ZFPs were subjected to domain confirmation through Hidden Markov Model (HMM) profiling, employing the HMMER toolset (https://www.ebi.ac.uk/Tools/hmmer/ accessed on 6 April 2025) and confirmed using conserved domain databases, including SMART (http://smart.embl.de/ accessed on 7 April 2025) and InterProScan (https://www.ebi.ac.uk/interpro/ accessed on 7 April 2025), to confirm the existence of specific C3H-ZFP motifs (zinc finger C-X8-C-X5-C-X3-H type and related motifs). The obtained sequences were then aligned and manually checked for their repetitions. In addition, the physico-chemical properties of the identified C3H-ZFPs, including theoretical isoelectric point (pI) and molecular weight (MW), were calculated by the ProtParam tool available on the ExPASy server [31]. To further elucidate the functional roles of the identified C3H-ZFP genes, we carried out gene ontology (GO) annotation and enrichment analyses using the BLAST2GO platform (https://www.blast2go.com accessed on 1 April 2025), which facilitated their categorization into key functional groups, containing biological functions, molecular, and cellular mechanisms.

### 2.3. Evolutionary Analysis

To investigate the evolutionary relationships between soybean C3H-ZFPs and their homologs in Arabidopsis, rice, and Medicago, a phylogenetic analysis was carried out using the MEGA7 software (https://www.megasoftware.net/ accessed on 1 April 2025) [32]. The analysis was established based on the maximum likelihood (ML) method. To evaluate the consistency and statistical confidence of the inferred phylogenetic clades, a bootstrap resampling analysis with 1000 iterations was performed. Bootstrap values were used to assess the support for each node within the phylogenetic tree [33].

### 2.4. Promoter Analysis, Expression Profiling, and TF Regulatory Networks of GmC3H-ZFP Genes

To examine the regulatory elements of GmC3H-ZFP genes, 2-kilobase (kb) upstream promoter regions were obtained from the Phytozome v13 genome database (https://phytozome-next.jgi.doe.gov/ accessed on 1 April 2025). The upstream promoter sequences were examined via the PlantCARE database to detect putative cis-regulatory elements potentially involved in transcriptional regulation [34]. RNA-seq data corresponding to GmC3H-ZFP genes under various tissue types were obtained from the Phytozome v12.1 database (https://phytozome-next.jgi.doe.gov/ accessed on 1 April 2025) for gene expression analysis. For the prediction of miRNAs (micro-RNAs), annotation analyses among GmC3H-ZFP genes was performed. All the GmC3H-ZFP CDS sequences were submitted as target candidates in the psRNATarget Server (https://www.zhaolab.org/psRNATarget/ accessed on 5 May 2025) and driven under default parameters for prediction of potential miRNAs in GmC3H-ZFP genes. The predicted miRNA and GmC3H-ZFP target gene interaction network was mapped by Cytoscape V 3.10 (https://cytoscape.org/ accessed on 1 April 2025). In addition, transcription factor (TF) regulatory networks associated with GmC3H-ZFP genes were predicted by submitting 1 kb upstream promoter sequences to the PlantRegMap database, using a *p*-value threshold of 1 × 10^−6^ [35]. Predicted TF binding sites were used to infer possible upstream regulators, and the resulting regulatory interactions were visualized as network diagrams in Cytoscape V 3.10, facilitating the identification of key transcriptional regulators potentially influencing GmC3H-ZFP gene expression.

### 2.5. Physical Mapping of GmC3H-ZFP Genes and Substitution Rates (Ka/Ks Ratio) Analysis

The genomic distribution of GmC3H-ZFP genes was determined based on annotated reference genome data for soybean. To visualize the chromosomal localization and investigate potential duplication events, the genes were mapped onto the soybean chromosomes using the TBtools software v2.323 (https://www.tbtools.com/ accessed on 1 April 2025). This mapping facilitated the identification of segmentally duplicated gene pairs, which were represented by colored connecting lines to indicate evolutionary relationships and possible shared ancestry. Multiple sequence alignments of the coding regions were performed using Clustal Omega (https://www.ebi.ac.uk/Tools/msa/clustalo/ accessed on 27 April 2025) to ensure accurate alignment of homologous gene pairs [36]. To investigate the evolutionary dynamics of the GmC3H-ZFP gene family, synonymous (Ks) and non-synonymous (Ka) substitution rates were calculated for each pair of duplicated genes, providing insights into the selective pressures acting on gene divergence. These estimates were obtained through a codon-based alignment using PAL2NAL, followed by maximum likelihood analysis implemented in the PAML software package 4.10.7 [37,38,39].

### 2.6. Statistical Analysis

The data are expressed as the mean ± standard deviation (SD) of three replicates. Statistical significance was evaluated using one-way ANOVA and Duncan’s multiple-range tests (*p* < 0.05) in IBM SPSS Statistics version 22.

## 3. Results

### 3.1. Identification and Classification of C3H-ZFPs in Soybean

**Figure 1:**
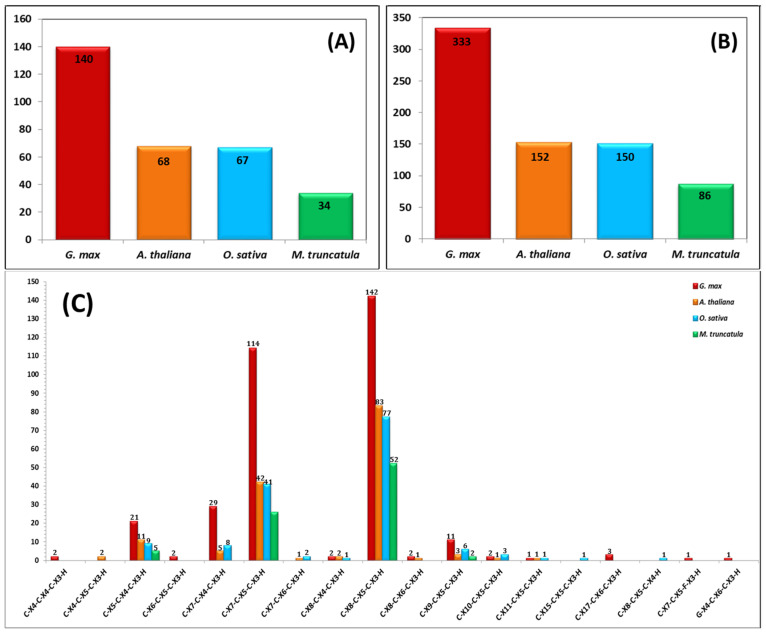
Characterization of C3H-ZFPs in soybean, Arabidopsis, rice, and alfalfa. C3H-ZFPs from *G. max*, *A. thaliana*, *O sativa*, and *M. truncatula* are shown. (**A**) Total number of C3H-ZFPs identified in each of the four plant species. (**B**) The distribution of C3H-ZFP domains per protein illustrates the species variation in domain copy number. (**C**) Comparative analysis of different types of C3H domains present in the C3H-ZFP protein families of *G. max*, *A. thaliana*. *O. sativa*, and *M. truncatula*.

A comprehensive identification of the C3H-ZF transcription factor members within the soybean genome is summarized in Table S1. A total of 140 C3H-ZFPs were identified and systematically named *GmC3H01* to *GmC3H140* based on their localization and gene IDs. There was a total of 140 retrieved non-redundant soybean C3H genes, which was far more than the total of C3H-ZFP genes found in *A. thaliana* (68), *O. sativa* (67), and *M. truncatula* (34) (Figure 1A). As shown in Figure 1B, a total of 333 C3H domains were identified in the GmC3H proteins, a number notably higher than those found in *O. sativa* (150) and *M. truncatula* (86) and approximately double that of *A. thaliana* (152). Each GmC3H protein contained between one and six C3H domains. Among the 140 putative C3H-ZFPs identified, 37 C3H-ZFPs contained a single C3H domain, 58 contained two C3H domains, 19 had three C3H-ZFP domains, seven had four C3H domains, 17 had five C3H domains, and two possessed six C3H domains while these domains were further classified into 14 C3H-ZFP subsets based on the variation in the specific conserved amino acid sequence and differences in the spacing between metal ligands within the motif “C-X_4–17_-C-X_4–6_-C-X_3_-H” (Figure 1C; Table S2). Analysis of the 140 soybean C3H-ZFP sequences revealed amino acid lengths ranging from 127 to 2020 residues, molecular weights spanning 14.47 to 223.38 kDa, and theoretical isoelectric points (pI) varying between 4.35 and 9.88. Gene ontology analysis demonstrated that a majority of GmC3H genes are associated with metal ion binding functions, predominantly localized within the nucleus, and implicated in a wide range of cellular and biological processes (Table S3).

### 3.2. Phylogenetic Analysis of the Classified Soybean C3H-ZFP Gene

**Figure 2:**
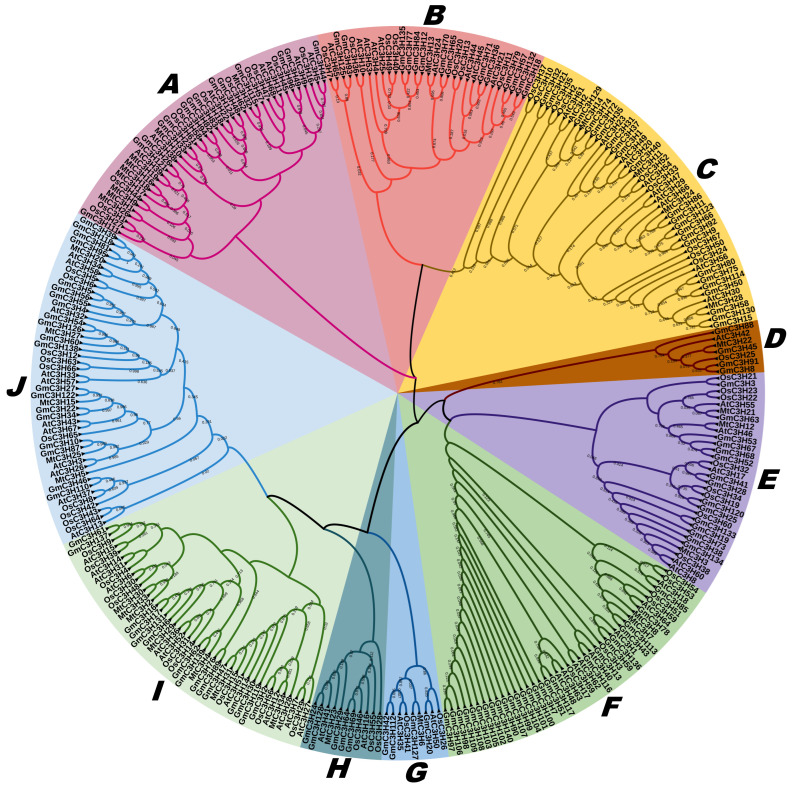
Phylogenetic classification of C3H-ZFP in soybean, Arabidopsis, rice, and Medicago. The tree was constructed using full-length C3H-ZFP amino acid sequences from *G. max* (*Gm*), *A. thaliana* (*At*), *O. sativa* (*Os*), and *M. truncatula* (*Mt*). The identified C3H-ZFPs were categorized into 10 distinct subgroups (**A**–**J**), each represented by a different color shade, reflecting their sequence similarity and potential functional divergence.

To elucidate the phylogenetic relationships among 140 C3H zinc finger proteins (C3H-ZFPs) in soybean, a comparative analysis was conducted using C3H gene sequences from *A. thaliana*, *O sativa*, and *M. truncatula*. A maximum likelihood (ML) phylogenetic tree was established based on multiple sequence alignments generated with the ClustalW algorithm, employed in MEGA 7.0 software (Figure 2). Based on the results, we categorized the GmC3H-ZFP genes into 10 major groups (A–J) as shown in Figure 2. Among these, the largest number of clustered genes was found in Group F, which contained 26 genes, followed by Group C, which comprised 23 genes. Group J contained 20 C3H-ZFP genes, while Groups E and I each contained 15 C3H-ZFP genes. Groups A and B each comprised 14 C3H-ZFP genes. Additionally, Group H contained five C3H-ZFP genes, and Groups D and G each had four genes. The analysis further identified orthologous relationships and highlighted a high degree of evolutionary conservation of C3H-ZFPs across the examined species.

Intron gain or loss represents a key evolutionary mechanism that contributes to gene structural diversity and complexity, playing a significant role in the functional diversification and divergence of multi-gene families throughout plant evolution [40]. To gain insights into the structural variation in C3H-ZFP genes, we examined the exon–intron organization of each soybean C3H-ZFP gene based on their genomic sequences, in relation to the phylogenetic groupings derived from Figure 2. The results indicated that closely related members were generally clustered together and exhibited highly similar exon–intron structural organization (Figure S1).

### 3.3. Chromosomal Distribution and Gene Duplication

**Figure 3:**
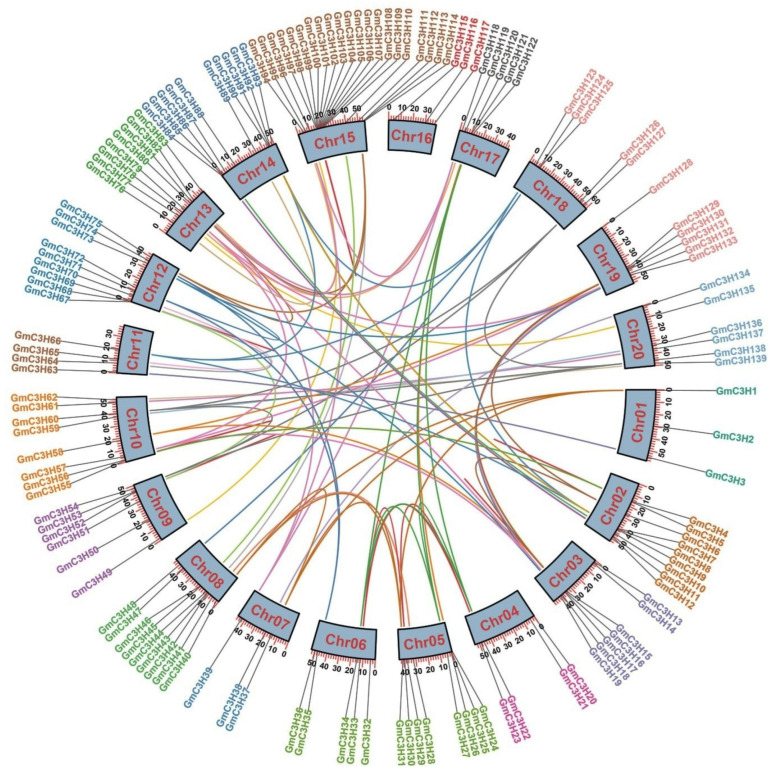
The spatial organization of C3H-ZFP genes across the 20 soybean chromosomes is illustrated, with each chromosome identified by its respective number (Chr1–Chr20). The gene names and their corresponding physical positions (Ml) are provided for each chromosome. Duplication events are represented by colored lines linking the duplicated gene pairs. Red lines specifically indicate tandem duplications.

To investigate a comprehensive overview of the genomic distribution of C3H-ZFP genes in soybean, we retrieved chromosomal position data for each known gene (Table S1) and constructed a physical map illustrating their positions across the soybean genome (Figure 3). Our results revealed that 139 out of 140 soybean C3H-ZFP genes (99.28%) were successfully mapped to the 20 chromosomes (Figure 3), while the remaining gene was positioned on an unanchored scaffold (Table S1). As shown in Figure 3, the C3H-ZFP genes were irregularly distributed throughout 20 chromosomes of soybean. Around 21 genes were present on Chr15, 10 genes on Chr14, and nine genes on Chr02, Chr08, and Chr12. In contrast, Chr1 and 16 harbored the fewest C3H-ZFP genes. Furthermore, duplication analysis revealed that 86 of these genes were duplicated, comprising eight tandemly duplicated pairs and 78 segmentally duplicated pairs (Table S4). Gene duplication events during evolution can lead to functional divergence, whereby duplicated genes may lose their original functions and potentially adopt novel functions. To assess the selective pressures acting on segmentally duplicated GmC3H-ZFP gene pairs, the ratio of non-synonymous (Ka) to synonymous (Ks) substitution rates was calculated (Table S4). The results showed that all duplicated GmC3H-ZFP gene pairs exhibited Ka/Ks ratios below one, indicating that these genes have undergone strong purifying selection following duplication and throughout subsequent species evolution.

### 3.4. Characterization of Additional Structural Motifs Apart from the C3H Zinc Finger Domain

To facilitate a more detailed classification of the 140 identified soybean C3H-ZFPs, we analyzed their full-length protein sequences using the SMART tool to detect the presence of any additional conserved domains beyond the characteristic C3H motifs. A total of 23 distinct domains, in addition to the C3H motifs, were identified. The 140 soybean C3H-ZFPs were classified into 16 major groups and subgroups based on the existence or nonexistence of these additional domains and their structural association (Table S5). The first major group consists of 48 members (34.28%) characterized solely by the presence of C3H domains, without any additional domains observed. Another major group comprises 27 members (19.28%), which are further subdivided into six subgroups, each characterized by the presence of RRM domain in addition to the C3H domains. In contrast, the remaining 14 groups consist of 1 to 18 members, each containing one to three additional known domains, which are predicted to mediate a variety of molecular functions, including protein–protein interactions (ANK, coiled-coil, RRM, JmjC, DFRP_C), protein binding (RING WD40 x 6, SET, PUG, CactinC_cactus, UBX), nucleic-acid binding (KH, G_patch, DEXDc, HELICc, PRVT_3), zinc ion binding (RING, Zf-U1), etc. All identified additional domains have also been reported in various plant species, such as *A. thaliana*, *M. truncatula*, *O. sativa*, *Solanum lycopersicum*, *Z. mays*, and others [9,10,11,12,13,41].

### 3.5. Promoter Region Analysis of GmC3H-ZFP Genes

**Figure 4:**
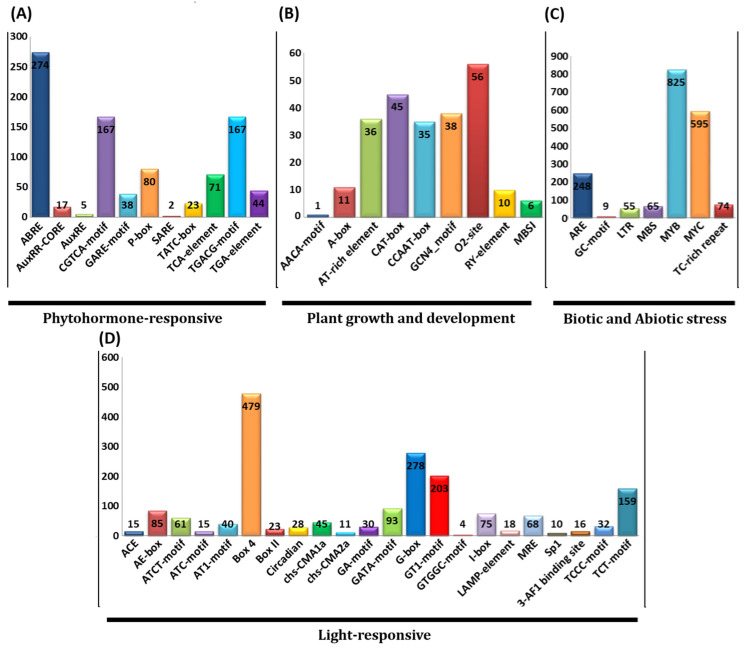
The cis-acting elements in the promoter regions of GmC3H genes were analyzed, with a colored histogram depicting their distribution across categories. The *x*-axis shows the categories, and the *y*-axis shows the frequency of elements in each category. (**A**) Phytohormone-responsive elements; (**B**) elements associated with plant growth and development; (**C**) biotic and abiotic stress-responsive elements; (**D**) light-responsive elements.

Cis-regulatory elements within the promoter regions of GmC3H genes were examined to elucidate the potential biological functions of these important genes. Specifically, 2 kb sequences upstream of the 5′-regions of GmC3H genes were obtained from Phytozome v13 and examined by the PlantCARE website. A total of 140 upstream regions of GmC3H genes were analyzed, revealing 4785 potential cis-regulatory elements. These elements were categorized into four primary categories: stress-responsive (39.10%), light-responsive (37.36%), and phytohormone-responsive (18.55%) elements and those associated with plant growth and development (4.97%) (Figure 4, Table S6). Among the identified cis-acting elements, three key light-responsive elements (Box-4, G-box, and GT1-motif), four phytohormone-responsive elements (ABRE, CGTCA, TGACG, and P-box) associated with abscisic acid, methyl jasmonate, auxin, and gibberellin signaling, four developmental-associated elements (O2-site, CAT-box, AT-rich, and GCN4 motif), and environmental stress-related elements (ARE, MYB, and MYC motifs) were frequently observed (Figure 4).

### 3.6. Spatial Expression Patterns of GmC3H-ZFP Genes in Various Tissues

**Figure 5:**
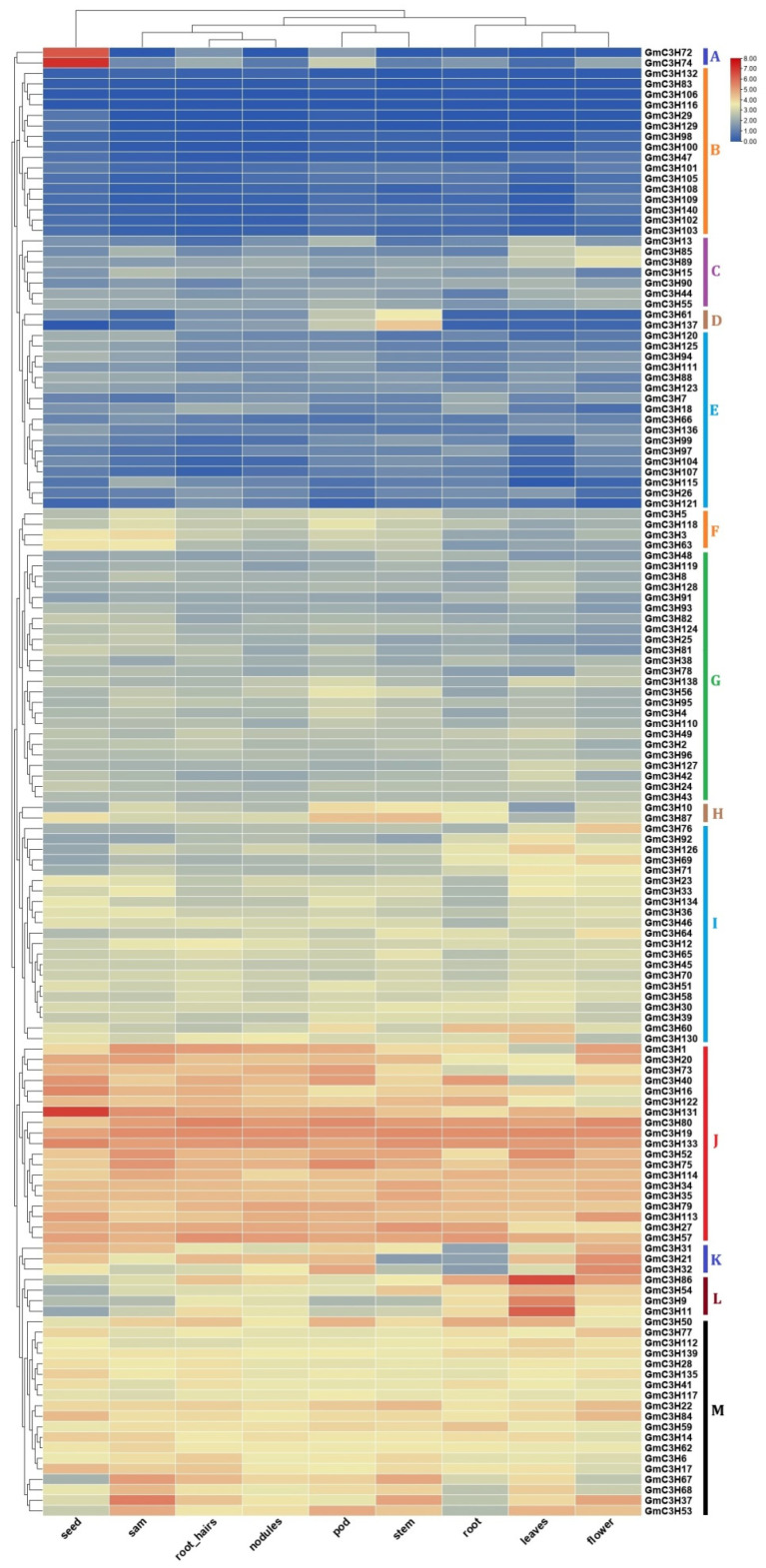
The expression profile of GmC3H-ZF genes across numerous tissues and organs is presented. The relative expression levels are shown as bars on the top side of the heatmap, with tissue or organ types present on the bottom side. The C3H-ZFP genes are listed on the right position of the heatmap. GmC3H genes are grouped into 13 distinct clusters (A–M), each represented by a different colored line.

The expression profiles of all 140 GmC3H genes were analyzed across multiple soybean organs and tissues such as seeds, roots, shoots, and leaves under various conditions, using publicly available data from the Phytozome database. The retrieved expression files (FPKM values) were log_2_-transformed, and a heat map was generated to visualize the expression patterns of the 140 GmC3H genes across various soybean tissues and organs (Figure 5). All GmC3H genes were classified into 13 distinct groups (A–M) as determined by their expression profiles (Figure 5). Group A comprised two GmC3H genes that were predominantly expressed in seed tissues. Group C comprised seven genes exhibiting pronounced expression in flowers and leaves, while Group D included two genes specifically expressed in the stem. Group F consisted of four genes with distinct expression across multiple tissues, including seed, shoot apical meristem (SAM), pod, and stem tissues. Similarly, Group H contained two genes with notable expression in both pod and stem tissues. Group K included three genes with prominent expression in flowers, pods, and seeds, while Group L consisted of four genes predominantly expressed in leaves. Groups J and M encompassed 19 genes that exhibited consistently high expression levels across all examined tissues. In contrast, Groups I and G comprised genes with moderate expression across all tissues. Groups B (16 genes) and E (17 genes) comprised members that were either very weakly expressed or not expressed at all in any of the examined tissues.

### 3.7. Expression Profiles of C3H-ZFP Genes Under on Salt and Cadmium Stress

**Figure 6:**
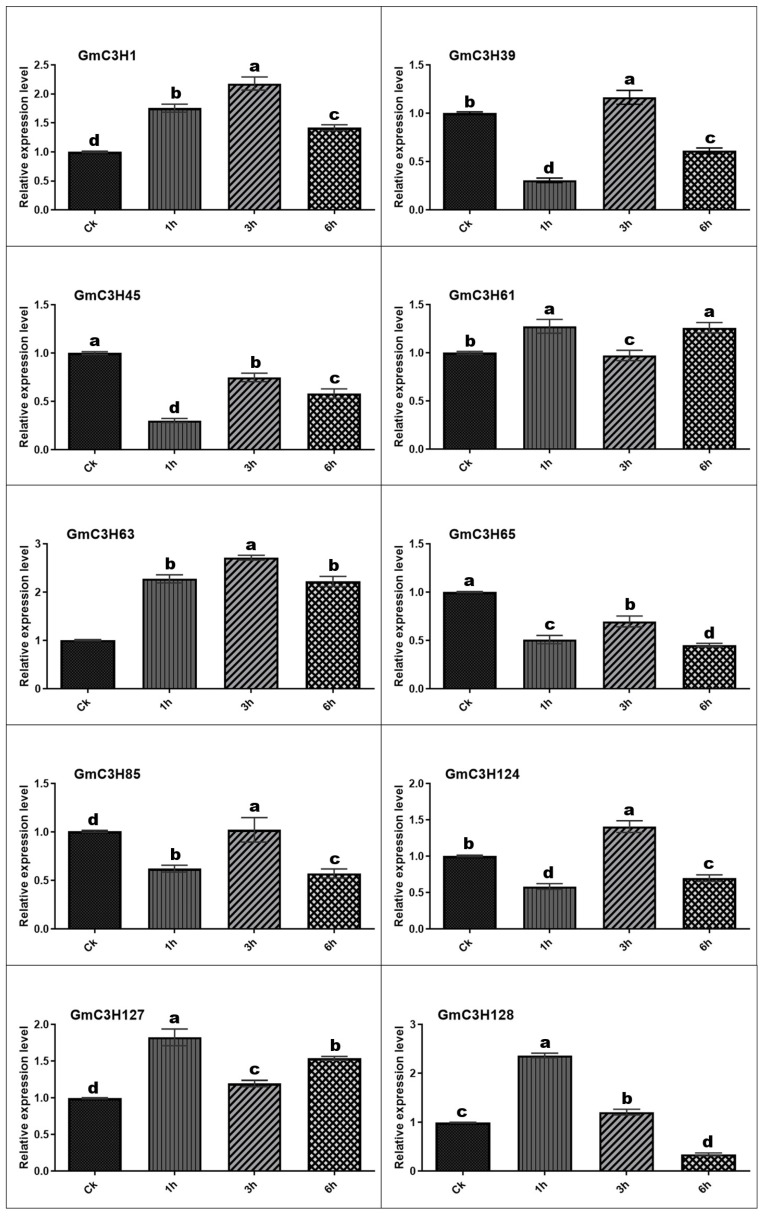
The expression of GmC3H genes under salt stress was examined by quantitative qRT-PCR. The *x*-axis indicates the time points, while the *y*-axis represents the relative expression levels. Duncan’s multiple-range test was employed to assess statistically significant variances in gene expression across different time intervals of salt exposure. Different letters above the bars indicate statistically significant variances, with a significance level at *p* < 0.05.

**Figure 7:**
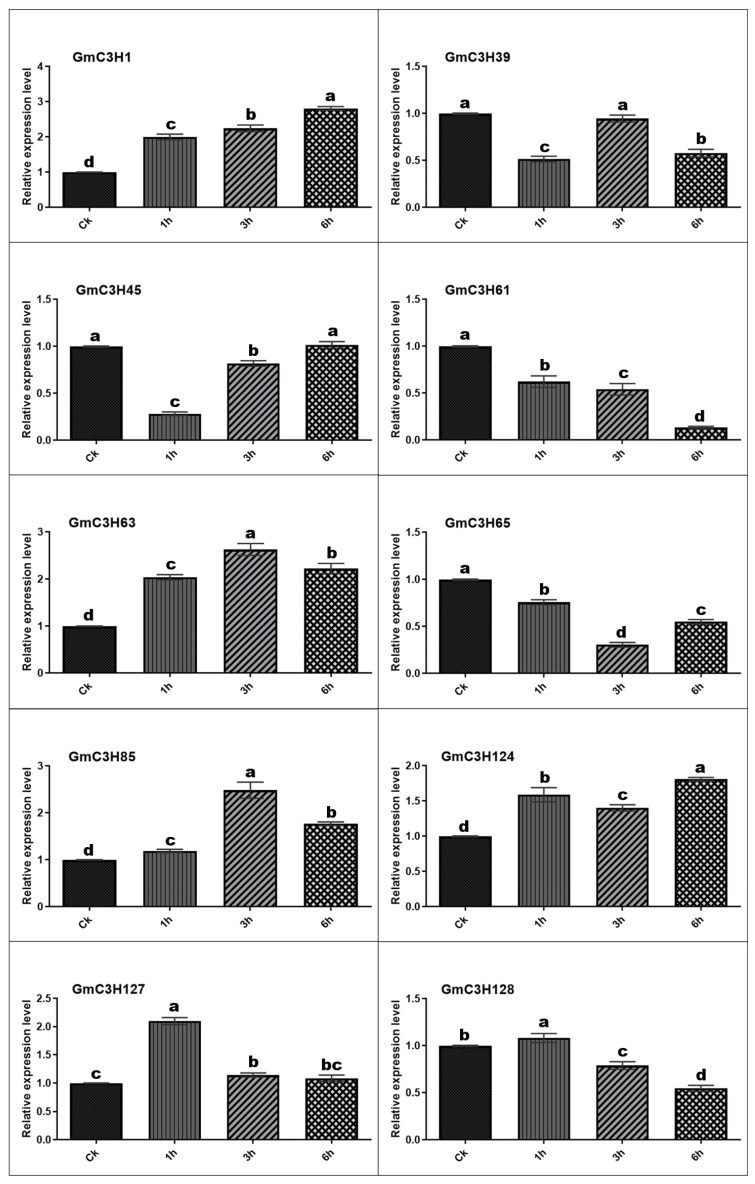
The expression of GmC3H genes under Cd stress evaluated by quantitative qRT-PCR. The *x*-axis indicates the specific time points, while the *y*-axis represents the relative expression levels. Duncan’s multiple-range test was employed to assess statistically significant variances in gene expression across different time intervals of Cd exposure. Different letters above the bars indicate statistically significant variances, with a significance level at *p* < 0.05.

To explore the potential involvement of GmC3H-ZFP genes in abiotic stress responses, qRT-PCR was performed to examine the expression dynamics of ten representative GmC3H-ZFP family members in soybean seedlings exposed to salt (NaCl) and cadmium (Cd) treatments. The ten genes were selected based on their phylogenetic classification, ensuring representation from different C3H-ZFP subgroups to reflect potential functional diversity within the gene family (Figure 2A–J). Transcript levels were measured at 1, 3, and 6 h after treatment and compared to untreated control (CK) samples (Figure 6 and Figure 7). Under salt treatment, all ten GmC3H genes exhibited differential expression across all examined time points compared with the control. Seven genes, *GmC3H1*, *GmC3H39*, *GmC3H61*, *GmC3H63*, *GmC3H124*, *GmC3H127*, and *GmC3H128*, were upregulated after different hours of salt stress exposure. Furthermore, *GmC3H45* and *GmC3H65* exhibited sustained downregulation at all examined time points (1 h, 3 h, and 6 h) under salt stress, whereas *GmC3H39, GmC3H85,* and *GmC3H124* were downregulated specifically after 1 and 3 h of treatment (Figure 6). In response to Cd stress, five GmC3H genes, namely *GmC3H1*, *GmC3H63*, *GmC3H85*, *GmC3H124*, and *GmC3H127*, exhibited significant upregulation. In contrast, two genes, *GmC3H61* and *GmC3H65*, were consistently downregulated relative to the control at 1, 3, and 6 h following Cd exposure. Similarly, *GmC3H45* was downregulated at 1 and 3 h, *GmC3H39* at 1 and 6 h, and *GmC3H128* at 3 and 6 h after treatment (Figure 7).

### 3.8. Analysis of Putative miRNAs and Their Targeting of C3H-ZFP Genes

**Figure 8:**
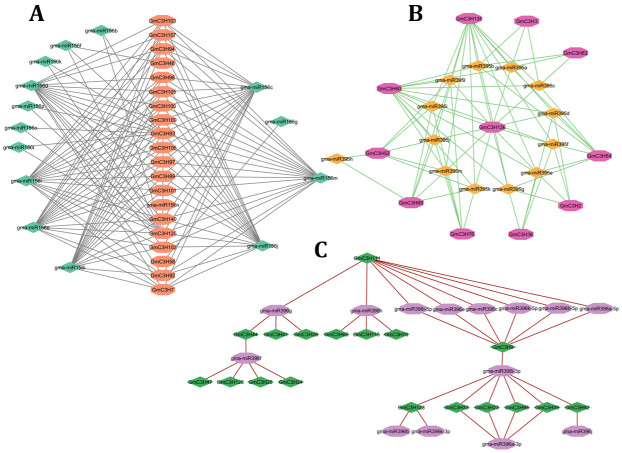
Network representation of miRNAs predicted to target GmC3H-ZFP genes. (**A**) GmC3H-ZFP genes targeted by the miR156 family. (**B**) GmC3H-ZFP genes targeted by the miR395 family. (**C**) GmC3H-ZFP genes targeted by the miR396 family. C3H-ZFP genes and their targeting miRNAs are depicted using distinct colored boxes, while different lines represent the interactions between the miRNAs and C3H-ZFP genes.

MicroRNAs have emerged as crucial post-transcriptional regulators that influence diverse aspects of plant physiology, including growth regulation, signal integration, and adaptive responses to environmental stimuli [42]. To investigate the potential microRNA-mediated regulation of GmC3H genes, we performed an in silico analysis and identified a total of 388 miRNAs belonging to 196 distinct gene families, which are predicted to target potential regulators of 140 C3H-ZFP genes (Table S7). Notably, the most enriched microRNAs predicted to target C3H-ZFP genes were visualized through an interaction network, highlighting their potential regulatory associations (Figure 8A–C). The analysis revealed that 20 C3H-ZFP genes were most frequently targeted by 14 gma-miRNAs belonging to the miR156 family (Figure 8A). Additionally, 11 C3H-ZFP genes were identified as targets of 13 gma-miRNAs from the miR395 family (Figure 8B). Furthermore, 11 genes were targeted by 10 gma-miRNAs from the miR396 family (Figure 8C). Consequently, future studies should experimentally validate the expression profiles of these predicted gma-miRNAs and their corresponding C3H-ZFP genes as well as their interaction, to clarify their important roles in key biological processes in the soybean genome.

### 3.9. Regulatory Network of C3H-ZFP Genes and Their Connected Transcription Factors

**Figure 9:**
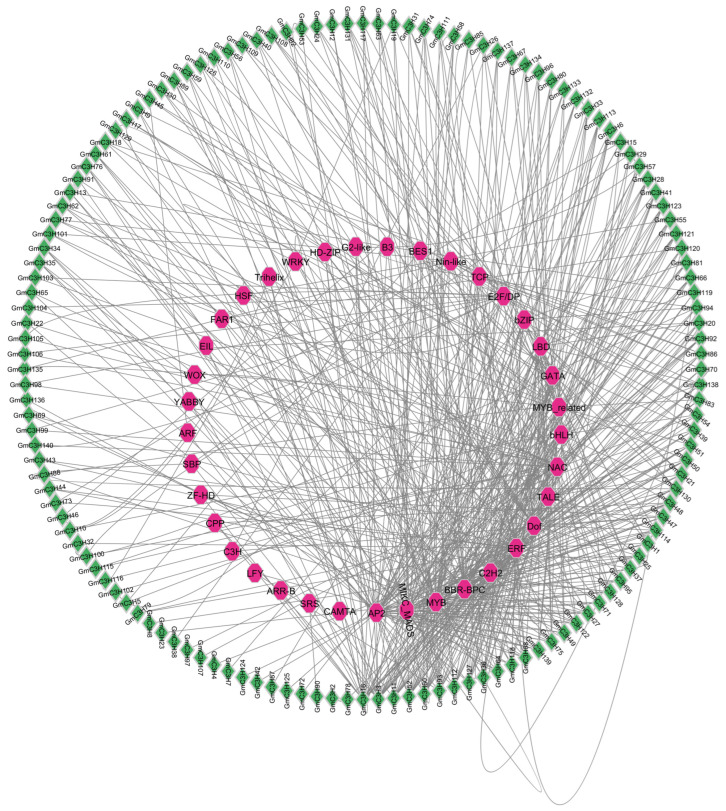
Exploratory analysis of the potential coexpression regulatory network of GmC3H-ZF genes. TFs are represented by pink circular nodes, while GmC3H-ZF genes are depicted as green nodes. The size of each node reflects the extent of interaction and enrichment among the genes and their corresponding TFs.

To gain insight into possible transcriptional regulation of C3H-ZF genes, a comprehensive in silico analysis revealed that 37 distinct TF families are predicted to be associated with 140 C3H-ZFP genes (Figure 9, Table S8). The results indicated 87 GmC3H members targeted by MYB transcription factor, with significant abundance, followed by AP2 (60 GmC3H members), MIKC_MADS (60 GmC3H members), BBR-BPC (50 GmC3H members), ERF (46 GmC3H members), C2H2 (46 GmC3H members), Dof (44 GmC3H members), TALE (33 GmC3H members), NAC (31 GmC3H members), bHLH (27 GmC3H members), and bZIP (17 GmC3H members) (Figure 9, Table S8).

## 4. Discussion

C3H-ZF genes, which are broadly conserved regulatory factors across multiple species, play crucial roles in plant development and stress responses by interacting with DNA, RNA, and proteins. This study aimed to conduct a comprehensive analysis of the C3H-ZFP gene family in soybean, with comparative insights drawn from the C3H-ZFP genes of the model plant *A. thaliana* [9]. This study elucidates the evolutionary lineage of the C3H-ZFP gene family and highlights the functional diversification of soybean C3H-ZFP genes. Our results offer new perspectives on the evolutionary history, structural features, and diverse expression profiles of C3H-ZFP genes in soybean, thereby enhancing the current understanding of this important gene family. Notably, we identified 140 C3H-ZFP members within the soybean genome, a number that exceeds those reported in *A. thaliana* [9]. The analysis substantiated the phylogenetic classification of the C3H-ZFP gene family into 10 distinct groups, emphasizing the heightened structural complexity and genetic diversity of soybean C3H-ZFPs in comparison to those found in Arabidopsis, rice, and alfalfa species [9,11,43].

Plant C3H-ZFP motifs display a wide spacing pattern classified into 14 different C3H motif subsets in soybean [9]. Similarly to that in other plant species, such as Arabidopsis, rice and Medicago, the C-X8-C-X5-C-X3-H motif represents the most prevalent C3H-ZF pattern in soybean (142, 42.64%). In comparison to other plant species, certain C3H zinc finger motifs are absent in soybean, whereas novel C3H motif variants seem to have emerged, signifying lineage-specific diversification within C3H-ZF gene family (Figure 1C). For instance, in comparison to Arabidopsis, C-X4-C-X5-C-X3-H and C-X7-C-X6-C-X3-H motifs are absent in soybean. Conversely, several novel motifs have been identified in soybean, including C-X4-C-X4-C-X3-H, C-X6-C-X5-C-X3-H, C-X17-C-X6-C-X3-H, C-X7-C-X5-F-X3-H, and G-X4-C-X6-C-X3-H (Table S2). Besides the C3H domains, soybean C3H proteins also contain various other important domains, including the RING, WD40, RRM, and KH domains (Table S5). Similar combinations of these domains have been studied in C3H proteins of other plant species, where the RRM and KH domains contribute to RNA processing, while WD40 repeats and the RING domain participate in protein–protein interactions and assembly of regulatory complexes [7,9,44,45,46,47,48].

The study revealed a distinct distribution of C3H-ZFP genes throughout all 20 chromosomes of soybean, implying that these genes have experienced numerous duplication events within the soybean genome [49]. The variable dispersal and density of C3H-ZFP genes across the soybean chromosomes suggest multifaceted evolutionary history, encompassing gene duplication events and subsequent functional divergence [50]. This phenomenon highlights the significance of gene duplication in evolution, potentially serving as the main source of novel gene functions. Gene duplication serves as a fundamental mechanism for the expansion of gene families within the genome, providing the basis for functional diversification and innovation [50]. The genome of ancient tetraploid soybean has been extensively shaped by two consecutive whole-genome duplication events, which have significantly influenced its genetic architecture and contributed to its evolutionary complexity [51]. Our genomic mapping analysis revealed that expansion of the C3H-ZFP genes in soybean is predominantly driven by segmental duplication; 78 pairs of C3H-ZFP genes are associated with segmental events, while eight pairs are related to tandem duplications (Figure 3). This imbalance suggests that large-scale chromosomal rearrangements, rather than local tandem amplification, play a major role in shaping the specified set of GmC3H-ZFP genes. To assess selective pressures on duplicated C3H-ZFP genes, we analyzed their Ka/Ks ratios. All duplicated C3H-ZFP gene pairs showed Ka/Ks values less than 1. This suggests that these gene pairs are evolutionarily conserved, likely due to their functionally significant roles and the selective pressure to maintain their integrity [52]. This finding is consistent with the principles of neo-functionalization, which propose that gene duplication can lead to the emergence of novel functions over the course of evolutionary diversification [53]. Therefore, the conservation and divergence perceived within the C3H-ZFP genes in soybean can be attributed to gene duplication events followed by functional diversification [53].

The upstream regulatory regions of the C3H-ZFP genes harbor important cis-regulatory elements comprising ABRE, CGTCA-motif, TGACG-motif, and P-box, which have previously been reported to be associated with phytohormonal signaling that regulate plant growth. Additionally, elements such as Box-4, G-box, and GT1-motif are linked to light response, while O2-site, CAT-box, AT-rich, and GCN4 motifs are related to developmental processes. Furthermore, stress-related motifs such as ARE, MYB, and MYC-motif suggest that C3H-ZFP gene expression may influenced by hormonal and environmental stress signals. These regulatory patterns are consistent with findings in Arabidopsis, as reported in previous studies [54,55].

The identification of 388 miRNAs from 196 families targeting 140 C3H-ZFP genes (Figure 8, Table S7) suggests extensive post-transcriptional regulation of this gene family. Especially, members of the miR156, miR395, and miR396 families target several key C3H-ZFP genes (Figure 8A); given their known roles in seed size regulation, this points to the potential contribution of C3H-ZFPs to seed development and yield-related traits [56,57]. Plant TFs play critical roles in diverse biological and molecular processes, including the regulation of gene expression, which in turn affects plant growth, development, and responses to environmental stresses [58]. In our analysis, MYB transcription factors were the most abundant TF family associated with the C3H-ZFP genes. Previous studies demonstrated that MYB TFs are key regulators of isoflavonoid biosynthesis in soybean [59]. The overrepresentation of MYB TFs in the predicted regulatory network of C3H-ZFPs suggests that these genes may participate in MYB-mediated pathways controlling secondary metabolism and stress adaptation. Following MYB, AP2 TFs were the next most abundant group. Members of the AP2/ERF family are known to participate in various aspects of plant growth, development, and stress response pathways [60,61,62]. In addition, several other TFs included MIKC_MADS, BBR-BPC, ERF, C2H2, Dof, TALE, NAC, bHLH, and bZIP (Figure 8, Table S8). Similarly to other regulatory genes, TFs such as Dof have also been implicated in enhancing plant biomass by promoting photosynthetic efficiency and starch accumulation [63].

Many previous studies have indicated that the majority of C3H-ZFPs function as TFs and are critically involved in regulating plant growth and developmental processes [64]. Expression profiling showed that 107 of 140 (76.42%) soybean C3H-ZFP genes were expressed in at least one of the nine tissues. Among these, about one-third exhibited consistently high expression across all tissues and one-third showed tissue-preferential expression in one to three tissues, while the remaining one-third displayed low expression levels in all tissues. These findings point to a possible functional diversification of the C3H-ZFP gene family in soybean, as reflected by their distinct tissue-specific expression patterns (Figure 5). Numerous members exhibiting tissue-specific expression patterns signify promising candidates for future functional characterization and may have potential application in the genetic improvement of oilseed crops.

Recent studies have demonstrated that the C3H-ZFP gene family plays a crucial role in plant responses to various abiotic and biotic stresses [14,15,16,65,66,67]. This study examined the expression profiles of ten phylogenetically selected soybean C3H-ZFP genes in response to salt and cadmium stress, aiming to identify their potential roles in stress response mechanisms. Our results demonstrated that all selected genes responded to both abiotic stress treatments, exhibiting differential expression patterns and varying magnitudes of response; while some genes were markedly upregulated, others showed downregulation over the course of the treatments (Figure 6 and Figure 7). These stress-responsive expression patterns, together with previously reported functional studies of C3H-ZFPs in other plant species, suggest that a subset of soybean C3H-ZFP genes may be involved in adaptation to adverse environmental conditions and represent promising targets for future functional characterization [68,69,70]. Although the ten genes belong to different subgroups and share close evolutionary relationships, their expression profiles vary significantly. This divergence in expression patterns specifies that these C3H-ZFP genes may be differentially associated with soybean responses to abiotic stress. Rather than confirming specific physiological functions, our results highlight these genes as stress-responsive candidates that need further experimental investigation. In particular, more functional studies such as loss and gain-of-function analyses will be required to clarify their precise roles in stress response pathways. Expanding future research to investigate a broader set of C3H-ZFP genes and additional abiotic stress conditions will contribute to a more comprehensive understanding of this gene family and support the identification of promising targets for the eventual genetic improvement of stress tolerance in oilseed crops.

## 5. Conclusions

In this study, we systematically identified and analyzed 140 C3H-ZFP genes in soybean, investigating their phylogenetic relationships, conserved GmC3H-ZFP domain, and chromosomal distributions. Phylogenetic analysis grouped the GmC3H-ZFP genes into 10 major clades, which were generally supported by similarities in conserved motifs and structural features. Gene duplication analysis indicated that segmental duplication was the primary force driving expansion of the GmC3H gene family. Notably, several C3H-ZFP genes were differentially expressed by salt and Cd stress, as validated through qRT-PCR analysis, and were predicted to participate in a broader co-expression network associated with stress responses. These results provide valuable insights into the evolutionary dynamics of the C3H-ZFP genes and highlight stress-responsive candidates that may be involved in abiotic stress adaptation, thereby laying a foundation for future functional genomics studies in soybean and other plant species.

## Data Availability

The original contributions presented in this study are included in the article/Supplementary Materials. Further inquiries can be directed to the corresponding authors.

## Supplementary Materials

The following supporting information can be downloaded at https://www.mdpi.com/article/10.3390/cimb48030287/s1.
