# Supplementary material for: Genome-Wide Identification and Characterization of C3H-ZFP Genes and Their Expression Under Salt and Cadmium Stress Conditions in Soybean
Source: Curr Issues Mol Biol. 2026 Mar 8;48(3):287. doi: 10.3390/cimb48030287 (PMC13025927; doi:10.3390/cimb48030287)

Figure S1. Graphical representation of the structural organization of soybean C3H-ZFP genes. The evolutionary relationship tree classification is shown on the left using the Maximum Likelihood (ML) method (A–J), followed by gene structure, where exons are shown in yellow, introns are represented by black lines, and UTRs are shown in green.

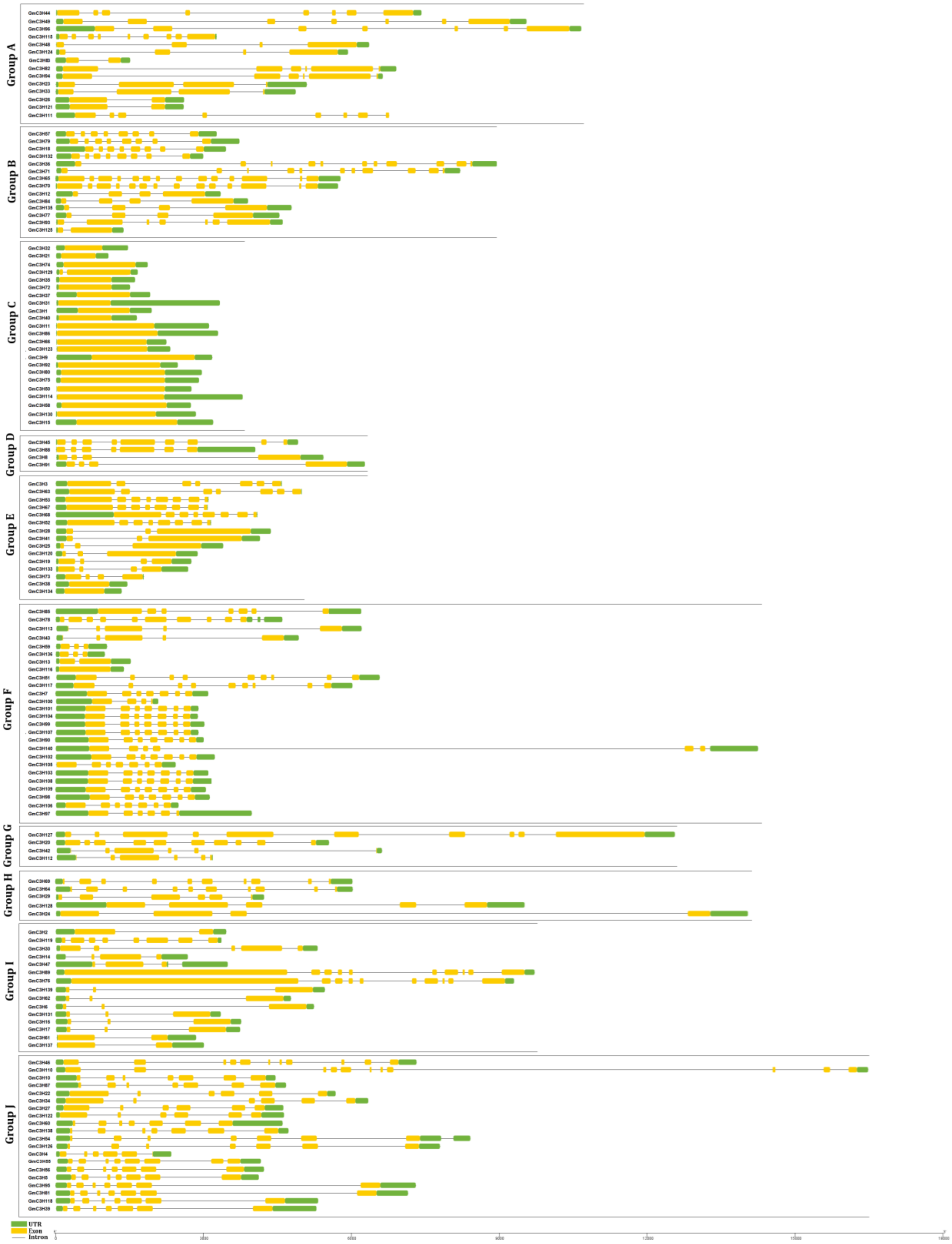

Supplement: Supplementary file 1 [file Figure S1.pdf › Figure S1.pdf]
